# Beyond Likert ratings: Improving the robustness of developmental research measurement using best–worst scaling

**DOI:** 10.3758/s13428-021-01566-w

**Published:** 2021-04-05

**Authors:** Nichola Burton, Michael Burton, Carmen Fisher, Patricia González Peña, Gillian Rhodes, Louise Ewing

**Affiliations:** 1 ARC Center of Excellence in Cognition and its Disorders, School of Psychology, University of Western Australia, Crawley, Australia; 2 School of Agriculture and Environment, University of Western Australia, Crawley, Australia; 3 School of Psychology, University of East Anglia, Research Park, Norwich, NR4 7TJ UK

**Keywords:** Face perception, Trust, Development, Children, Measurement, Best-worst scaling

## Abstract

Some of the ‘best practice’ approaches to ensuring reproducibility of research can be difficult to implement in the developmental and clinical domains, where sample sizes and session lengths are constrained by the practicalities of recruitment and testing. For this reason, an important area of improvement to target is the reliability of measurement. Here we demonstrate that best–worst scaling (BWS) provides a superior alternative to Likert ratings for measuring children’s subjective impressions. Seventy-three children aged 5–6 years rated the trustworthiness of faces using either Likert ratings or BWS over two sessions. Individual children’s ratings in the BWS condition were significantly more consistent from session 1 to session 2 than those in the Likert condition, a finding we also replicate with a large adult sample (N = 72). BWS also produced more reliable ratings at the group level than Likert ratings in the child sample. These findings indicate that BWS is a developmentally appropriate response format that can deliver substantial improvements in reliability of measurement, which can increase our confidence in the robustness of findings with children.

Psychologists are widely calling for more open and rigorous research practices in response to concerns about reproducibility within the discipline (Open Science Collaboration, 2015). Unfortunately, some of the measures put forward to help optimize key elements of the scientific process (see Munafò et al., 2017) can be difficult for individual developmental and clinical researchers to implement. For example, it is not always possible to bolster the robustness of conclusions with increased statistical power (large sample sizes, trial numbers) and internal replication when access to participants is constrained, and/or limited attention spans prohibit lengthy testing sessions.

In such cases, the best target for optimizing measurement may be improving task *reliability*. Error variance inflates observed variability, so reducing error can improve effect size estimates and therefore improve statistical power (Kanyongo et al., 2007). Data from children (cf. adults) is at elevated risk of being ‘noisy’ due to task-specific variables and/or more generic influences on performance such as attenuated ability to concentrate and avoid distractions, understand detailed instructions, and accurately execute required motor responses (see McKone et al., 2012). Thus, any methodological advances that can increase reliability of measurement are of great utility for developmental research.

Here we highlight one approach to improving the reliability of behavioural response measurement from children: updating the response format. We do so in the context of quantifying participants’ preferences for, or impressions of, a set of stimuli. Such measurement of preferences/impressions is common to many psychological fields. For example, researchers might need to assess participants’ judgments of the valence of words (Hollis, 2018), their views about the acceptability of the outcomes of a moral dilemma (Paxton et al., 2012), or how well they believe a set of personality descriptors apply to them (Goldberg, 1999). This type of measurement is also utilized when researching the work preferences of high achievers (Trank et al., 2002), the perceived attractiveness of different body types across cultures (Singh, 2004), or how trustworthy a person’s face appears to others (Oosterhof & Todorov, 2008).

Such measurements of participants’ subjective impressions are typically recorded using Likert ratings. Participants communicate their impression of a trait (e.g., trustworthiness) to the experimenter by translating their percept onto a numbered scale that ranges from 1 to some upper limit: anything from 3 to 100. These Likert ratings are simple to administer and score. Critically, however, there are a number of potential problems associated with their use, particularly in studies with children. First, they rely on the participant’s understanding the concept of a number line. This can be a problem for younger children (although measures can be taken to improve understanding, such as adding verbal labels to all points on the scale: Mellor & Moore, 2014). Children also tend to be extreme in their responses, favouring the two end points of the scale (Chambers & Johnston, 2002). Such a profile compresses items together at either end of the scale, concealing any differences between impressions of the items. Finally, the use of Likert scales can be cognitively demanding. Participants must retain a consistent calibration of the scale from item to item: a face rated as a ‘4’ on trustworthiness at the start of the task should be as trustworthy in appearance as a face given the same rating at the end of the task. To achieve such consistency, participants may rely upon remembering the responses that they have made throughout the task, and comparing the current item to previously rated items. Differences in short-term memory capacity and/or the availability of experience-based cognitive procedures (strategies) are likely to make it difficult for young children to maintain such calibration of the scale. Together, these difficulties with Likert ratings are likely to introduce error to responses, reducing measurement reliability.

In the current paper, we investigate an alternative method for quantifying participants’ preferences or impressions, known as best–worst scaling (BWS) (Louviere et al., 2015). The structure of the BWS method allows us to avoid many of the issues associated with Likert ratings. There are several forms of BWS; here we use BWS Case 1. In each trial of case 1 BWS, participants view a subset of items from the total set to be rated, and select the ‘best’ and ‘worst’ items. For instance, we might ask participants to choose the most trustworthy and least trustworthy from each subset of faces. The same face appears in multiple different trials across the task. By examining when a face is chosen as most trustworthy and when it is chosen as least trustworthy, we can determine the latent ‘perceived trustworthiness’ score for each face. One simple method for scoring a BWS task is to count the number of times a face is selected as ‘most trustworthy’, and subtract the number of times a face is selected as ‘least trustworthy’ (each item is presented equally often, so there is no need to normalize scores by the number of presentations). Notwithstanding the contribution of any probabilistic noise, the most trustworthy face in a set should be selected as most trustworthy in all trials in which it appears, achieving the highest possible score, and the least trustworthy face in a set should be selected as least trustworthy in all trials in which it appears, achieving the lowest possible score. All other faces will fall between these two values.

BWS has already been demonstrated to improve reliability for adult respondents compared to Likert ratings. When judging the attractiveness or distinctiveness of faces (Burton et al., 2019), and when judging verbal statements, (Kiritchenko & Mohammad, 2017), BWS produces both more reliable group-level ratings and higher consistency in individual participants’ ratings across sessions than the Likert method. The studies noted here made use of crowdsourcing platforms for their recruitment: across three studies, Burton et al. (2019) tested 924 Amazon Mechanical Turk workers (431 male) who were Caucasian and resided in the USA, with a mean age of 36.2 years; Kiritchenko and Mohammad (2017) used CrowdFlower, noting only that their workers were required to be native English speakers from the USA.

There are several advantages associated with the BWS approach that can explain BWS’s superior reliability. The forced-choice nature of the task encourages participants to distinguish items that differ in perceived trustworthiness. Participants cannot compress items together within a narrow range of responses, as is the risk with Likert ratings. However, where two items genuinely cannot be distinguished, they are equally likely to be selected as ‘most trustworthy’ in a trial, and therefore should ultimately receive very similar scores. Importantly, BWS requires that each response be made only in terms of the current subset of items. Participants do not need to remember their responses in previous trials in order to give meaningful responses. We expect that these advantages will be particularly relevant for child participants. Additionally, the ‘most/least’ format does not require children to be able to express subtle differences in degree. Thus, we propose that this less demanding format is particularly well suited for use with young children.

Here, we test whether children aged 5–6 years can successfully rate their impressions of facial trustworthiness using the BWS format, and whether this young population also shows improved reliability for BWS compared to traditional Likert ratings. We opted to investigate facial trustworthiness because of the social importance of these attributions. Adults are known to automatically form trustworthiness impressions from faces with a high degree of consensus, which can have powerful consequences across a range of contexts (see Olivola et al., 2014; Todorov et al., 2015). Perhaps unsurprisingly, given applied relevance to safeguarding and ‘stranger danger’ awareness, the development of children’s perceptions of trustworthiness has also captured particular research attention. Evidence supports some degree of perceptual sensitivity to facial trustworthiness cues from impressively early in development (see EEG studies with infants (e.g., Jessen & Grossmann, 2016, 2017). By 3 to 4 years of age, children are able to explicitly discriminate between trustworthy-looking and untrustworthy-looking individuals using categorical labels (e.g., ‘nice vs not nice’, Cogsdill et al., 2014) and Likert-style rating scales (Caulfield et al., 2016; Ewing et al., 2015; Ma et al., 2016).

Although we know that children are sensitive to cues of facial trustworthiness, the absence of robust, developmentally appropriate measures has precluded detailed investigations of important questions relating to these perceptions. For example, existing studies have shown poorer sensitivity to facial trustworthiness cues in younger children compared to older children (Caulfield et al., 2016; Ma et al., 2016). Given the measurement issues associated with Likert ratings, it is currently difficult to determine to what extent this deficit is driven by problems with scale use, as opposed to differences in the children’s impressions themselves. If there are developmental differences in trustworthiness impressions, a more reliable measure would also enable us to determine the visual cues that differentially drive children’s impression formation—for instance, there is currently conflicting evidence as to whether children use subtle facial expressions as a cue to trustworthiness (Ewing et al., 2019; Mondloch et al., 2019). Furthermore, our ability to measure the extent to which there are individual differences present *within* age groups also depends on the ability to reliably measure children’s impressions.

In the present study, participants used either the BWS or Likert method to rate the trustworthiness of a set of faces twice in two sessions. We predicted that an individual child’s responses to the same faces would correlate more strongly from one testing session to the next when measured with BWS as compared to Likert ratings. We also predicted that group-level ratings calculated by taking the average rating for each face across participants would be more reliable (as measured by Cronbach’s alpha) when measured with BWS as compared to Likert ratings. We also tested a group of adult participants on the same tasks. Based on the findings of Burton et al. (2019) and Kiritchenko and Mohammad (2017), we also expected to find the same pattern of improved individual and group-level reliability for this adult group.

## Methods

### Participants

Participants were 73 children aged 5–6 years old, and 72 adult psychology undergraduates (a population with the typical large percentage of females), who were randomly allocated to one of two conditions, BWS or Likert, see Table 1 for details. Five additional children were tested, but four were excluded due to technical errors resulting in unusable data, and one was excluded after informing experimenters of having made deliberately incorrect responses. Children received stickers and a certificate, and adults were provided with course credit. Adult participants provided written informed consent. The parents of child participants provided written informed consent, and children provided verbal assent to participate. The study was granted ethical approval by the University of East Anglia under project name ‘Best Worst Scaling as an alternative to Likert ratings in children’s face perception’ (ref. 2017-0198-000848) and by the University of Western Australia under project name ‘Understanding Face Perception’ (ref. RA/4/1/2323), and conforms to the Declaration of Helsinki.

**Table 1:** Age and sex for adult and child participants in each condition

	Children		Adults	
	M Age (SD)	N (% Male)	M Age (SD)	N (% Male)
BWS	6.0 years (0.3 years)	38 (44.7%)	26.2 years (12.5 years)	37 (24.3%)
Likert	6.1 years (0.3 years)	35 (45.7%)	28.5 years (13.5 years)	35 (22.9%)

Age did not significantly differ between conditions for either age group (children *t*(71) = 0.84, *p =* .403; adults *t*(70) = 0.752, *p* = .455)

### Sample size justification

A power analysis was carried out using the pwr package in R Version 3.5 (R Core Team, 2013). This analysis was based on the results of a previous study conducted by Burton et al. (2019). These authors observed a difference of d = 0.68 between the self-consistency correlations in their adult BWS (r = .66) and Likert (r = .53) groups for ratings of attractiveness, and d = .73 between adult BWS (r = .76) and Likert (r = .63) groups for ratings of trustworthiness. We based our power analysis on the more conservative estimate (d = .68).

### Stimuli

Stimuli were 30 male faces taken from the 10k US Adult Faces Database, a collection of ambient face images taken from Google image searches. Images in this database are oval masked around the face to minimize background information. We restricted our stimulus set to Caucasian non-celebrities who were forward-facing with direct gaze, and screened for acceptable image quality. The 10K US database includes trait ratings for a subset of these faces; we pseudorandomly selected images based on mean trustworthiness rating to cover the full range of perceived trustworthiness.

### Procedure

Child and adult participants both followed the same testing procedure, i.e., the same games/tasks with the same instructions. Children completed testing in a quiet room at school. Most adults were tested in a quiet room at the university, but due to social distancing restrictions three adults (one in the BWS condition, two in the Likert condition) completed their second session online, and five (all in the Likert condition) completed both sessions online. Participants were introduced to a child-friendly game in which they had to help Zeb the Alien learn about trustworthiness. Following the approach taken in previous developmental studies of trustworthiness perception (e.g., Caulfield et al., 2014; Ewing et al., 2015), the first session began by introducing participants to the following definition of trustworthiness:

*A person who is trustworthy is someone who is very honest with you, someone who is reliable and will keep his or her promises to you, and someone who will keep a secret if they need to.*

Participants then completed a comprehension check to ensure that they understood this operationalization of the construct. Finally, participants completed one of two perceptual tasks, depending on whether they were in the BWS or Likert condition.

In each trial of the Likert task, participants rated faces on a five-point scale that was constructed to be as child-appropriate as possible (see Cooper et al., 2006). The numbers 1 to 5 were shown with cups of increasing size to indicate increasing quantity, and each point was anchored with a verbal label (1: ‘very untrustworthy’, 2: ‘a little untrustworthy’, 3: ‘so-so or ok trustworthiness’, 4: ‘fairly trustworthy’, and 5: ‘very trustworthy’). Participants began by using this Likert scale to rate three cartoon faces presented individually, before moving on to rate the 30 stimulus faces. These 30 faces were rated in the same fixed order for all participants.

On each trial of the BWS task, participants were shown five faces and asked to select the most trustworthy and least trustworthy faces out of the set. Participants began with two practice trials, each showing a set of five cartoon faces, before moving on to the stimulus faces. Each face was shown in five trials, giving a total of 30 trials. All participants saw the same sets of faces, presented in the same order. Faces were arranged into subsets using Sawtooth (Sawtooth Software, 2009), which was also used to present the BWS trials.^1^

During their second testing session, participants completed exactly the same task as they had completed in the first session. Children completed session 2 on the day following session 1. Adults completed the two sessions between 1 and 11 days apart; number of days between sessions did not differ significantly between BWS (*M* = 2.4 days, *SD* =2.7 days) and Likert (*M* = 1.5 days, *SD* = 1.0 days) conditions, *t*(70) = 1.85, *p* = .068, Cohen’s *d* = .44.

## Results

BWS scores were determined using the simple counts method^2^: for each face, we took the number of times that a given participant had chosen it as ‘most trustworthy’ in a testing session, and subtracted the number of times that the participant had chosen it as ‘least trustworthy’ in that testing session. This yielded a single score or ‘rating’ representing the perceived trustworthiness of that face. We calculated these BWS scores separately for each session, for each participant.

**Fig. 1:**
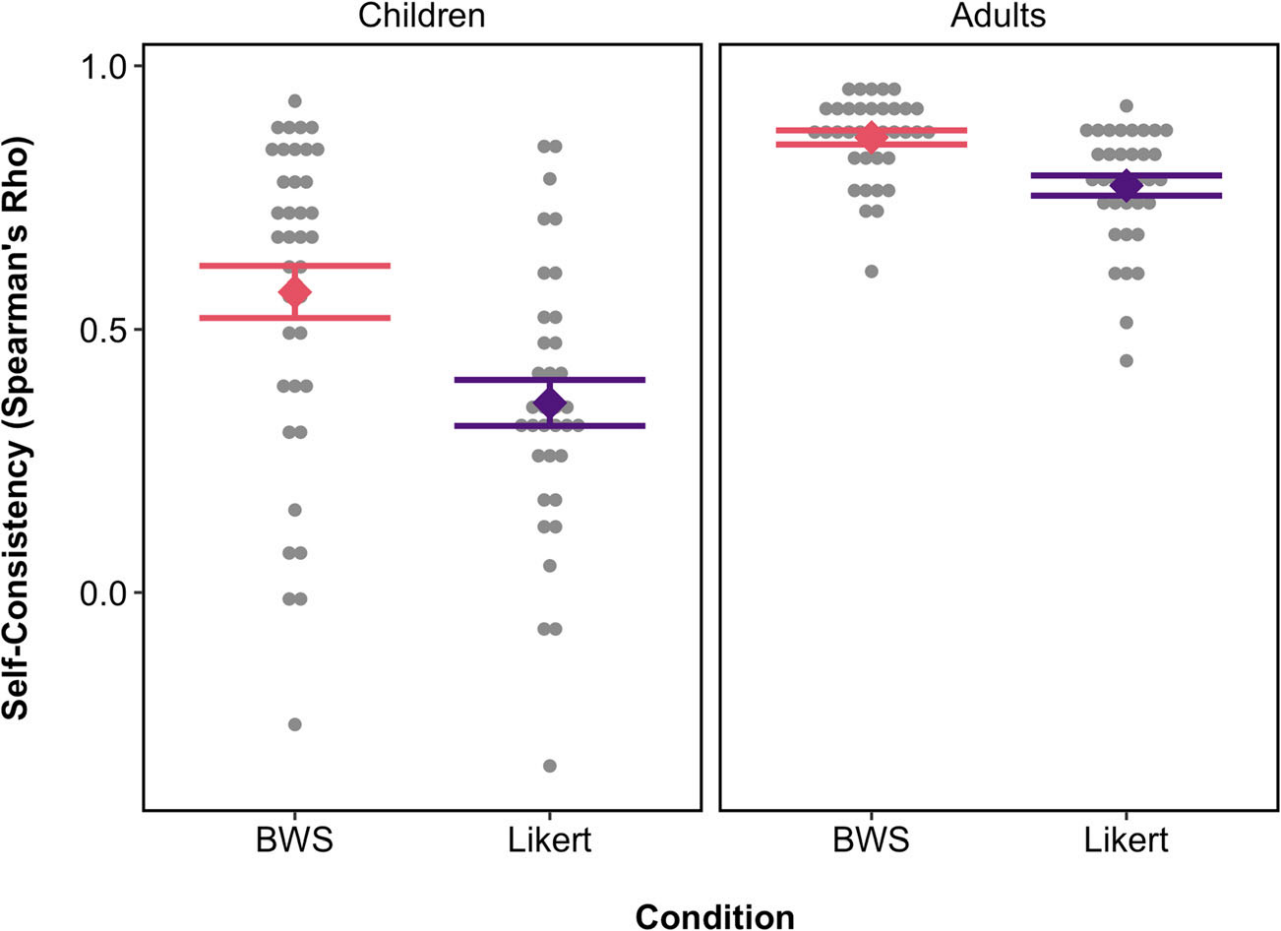
Self-consistency for children and adult participants in the BWS and Likert conditions. Self-consistency was measured as the Spearman’s rho correlation between each participant’s ratings of the faces at session 1 and session 2. Grey points show individual participants’ self-consistency scores; coloured points show mean self-consistency for each age group and condition. Bars show ±1 SE

For a measure of the reliability of individual-level ratings for each of the two methods, we calculated a self-consistency score for each participant. The self-consistency score was the Spearman’s rho correlation between each participant’s judgments of the 30 faces in session 1 and their judgments of the same 30 faces in session 2. A lower self-consistency score indicates more variability in responses between the two sessions. Because correlation coefficients are bounded, we Fisher-transformed the self-consistency scores for parametric analysis (Fisher, 1915). Figure 1 shows untransformed scores for ease of interpretation.

Children showed higher self-consistency in the BWS condition than in the Likert condition. An independent-samples *t* test indicated that this difference was statistically significant, *t*(71) = 3.53, *p* = .001, Cohen’s *d* = 0.54. One adult was identified as an outlier (BWS condition: untransformed self-consistency = 1) and was removed from this analysis. The remaining adult group showed the same pattern of significantly higher self-consistency in the BWS condition than the Likert condition, *t*(69) = 4.39, *p* <.001, Cohen’s *d* = 0.58.

We also examined the group-level reliability of the two methods. Participant ratings can be averaged across the group to give a single rating for each face. The reliability of these group-level scores can be measured with Cronbach’s alpha, with participants entered as ‘items’ in the analysis (Berry & Wero, 1991). Calculated this way, Cronbach’s alpha estimates the correlation between this group’s ratings and the ratings of another group of the same size. This measure can also be thought of as an indication of the consistency of ratings across individuals—the more consistent the ratings are across individuals, the more reliable the group-level ratings will be. Because the number of ‘items’ in the analysis influences Cronbach’s alpha, sample size must be comparable for a comparison to be made between alpha values. For both age groups there were 35 participants in the Likert condition, so we took 50 random samples of 35 participants from the BWS condition, calculated alpha for each sample and found the mean value. For children, group-level reliability was higher in the BWS condition (*alpha* = .88) than in the Likert condition (*alpha* = .81). For adults, the group-level reliability was more comparable between the two methods (*BWS alpha* = .95, *Likert alpha* = .98). We tested the significance of these differences using the W test described by Feldt and Kim (2006). Group-level reliability was not significantly different between conditions for the children (*W*_27,28 =_ 1.58, *p* > .25) but was significantly higher for Likert than BWS scores for the adults (*W*_27,28 =_ 2.5, *p* < .25).

## Discussion

The current results indicate that BWS is an advantageous method for measuring the trustworthiness impressions of children as young as 5–6 years. Our test–retest reliability analysis confirms empirically, for the first time, that children’s trait impressions are not optimally measured with Likert ratings. We identified more consistent responses from both child and adult participants when perception was measured using BWS than when using a more traditional five-point rating scale. This finding suggests that Likert ratings include response error that can be limited/avoided by utilizing BWS. We also observed greater reliability of children’s mean ratings for individual faces at the group level for BWS compared to Likert.

BWS may be less vulnerable to the issues that can introduce error into children’s Likert ratings, such as difficulties in expressing differences in degree on a number line, tendency to prefer extreme responses (i.e., the end points of the scale), and difficulty maintaining perceptual and/or conceptual calibration over the duration of the task. We observed this advantage for BWS even when comparing against a developmentally appropriate version of the Likert scale, which included verbal anchors for all points (Mellor & Moore, 2014) and images of cups of various sizes to emphasize the concept of increasing amounts of trustworthiness (see Cooper et al., 2006).

Targeting improvements in reliability is vital for developmental research, where experimental power is often limited by sample sizes and the short attention spans of participants. Decreasing measurement error will increase effect sizes, boosting power and therefore our confidence in research findings. In the face of the current ‘replication crisis’, the reliability of methods should be considered carefully, and tools such as BWS that can improve reliability should be employed where possible. Additionally, where expected effect sizes are small, gains in reliability and experimental power can increase our ability to find an effect at all. For instance, in future studies seeking to establish the cues that contribute to children’s facial trustworthiness impressions, the reliability with which those impressions are measured places a ceiling on the possible correlation between cues and impressions.

We have successfully used BWS to capture the trustworthiness impressions of children as young as 5, and there is considerable scope to extend the current study and explore a range of judgments, preferences, and experiences in even younger age groups. Future research should also consider the utility of BWS for testing populations with cognitive limitations that may also make utilizing a Likert method particularly difficult, but would have a more reliable capacity to choose the ‘best’ and/or ‘worst’ from a set of options. Since performance does not rely on memory of previous trials, BWS should also allow testing to be broken up with sufficient breaks to allow more data to be collected from participants with limited cognitive resources. We have shown here that BWS yields a reliable and valid dependent variable that could offer new insights into the perception and judgements of special populations.

In conclusion, we have shown that BWS is an advantageous method for measuring the judgements of children as young as 5 years old. When compared with the standard (‘classic’) Likert response scale approach to measurement of participants’ perception of trustworthiness, BWS produced greater reliability for both individual-level and group-level scores in children, and for individual-level trustworthiness scores in adults. We propose that future studies can benefit profoundly from the application of BWS to the measurement of questions that could not be addressed with Likert methods. The use of this efficient and user-friendly approach can reduce measurement error and increase reliability, making it a useful tool both for studying individual differences in children and, crucially, for improving the replicability of experimental findings in developmental research.

The datasets generated during and/or analysed during the current study are available in the Open Science Framework repository, https://osf.io/2jpx3/?view_only=1c5a748ebfff486f868ec971c06a51be. No part of the study procedures or analyses was pre-registered prior to the research being conducted.

## Footnotes

1 We used Sawtooth Software’s Lighthouse Studio (Sawtooth Software, 2009) to design and present our BWS task, since it provides a streamlined process. However, the same task could also be designed using other software packages, e.g., the supportBWS package for R (Aizaki et al., 2014), and presented in any online or paper medium. See Additional Files associated with Burton et al., 2019 for helpful materials including “A beginners guide to the process of designing, running and scoring a Best–Worst Scaling Task’, annotated R scripts and supporting files.

2 This is a well-established scoring method for BWS responses (Louviere et al., 2015, sec.2.3.2). Alternative scoring methods are also used. For instance, Lipovetsky and Conklin (2014) developed a method of adjusting the simple count scores logarithmically that can improve estimates under certain conditions. Scores can also be estimated using conditional logistic regression models (Louviere et al., 2015, sec.2.3.3). However, these scoring methods are only appropriate when no item is ever selected as ‘best’ in every trial in which it appears (or ‘worst’ in every trial in which it appears). This problem is less often encountered when BWS is used to find scores for items using the responses of an entire group of people, but is more likely to occur when scoring the responses of individual participants, as in the case of this study.

## References

[CR1] Aizaki, H., Nakatani, T., & Sato, K. (2014). *Stated preference methods using R*: Chapman and Hall/CRC.

[CR2] Berry DS, Wero JL (1991). Accuracy in face perception: A view from ecological psychology. Journal of Personality.

[CR3] Burton N, Burton M, Rigby D, Sutherland CAM, Rhodes G (2019). Best-worst scaling improves measurement of first impressions. Cognitive Research: Principles and Implications.

[CR4] Caulfield F, Ewing L, Bank S, Rhodes G (2016). Judging trustworthiness from faces: Emotion cues modulate trustworthiness judgments in young children. British Journal of Psychology.

[CR5] Caulfield, F., Ewing, L., Burton, N., Avard, E., & Rhodes, G. (2014). Facial Trustworthiness Judgments in Children with ASD Are Modulated by Happy and Angry Emotional Cues. *PLoS ONE, 9*(5). 10.1371/journal.pone.0097644 10.1371/journal.pone.0097644 PMC4039438 24878763

[CR6] Chambers CT, Johnston C (2002). Developmental differences in children's use of rating scales. Journal of Pediatric Psychology.

[CR7] Cogsdill EJ, Todorov AT, Spelke ES, Banaji MR (2014). Inferring character from faces: A developmental study. Psychological Science.

[CR8] Cooper PA, Geldart SS, Mondloch CJ, Maurer D (2006). Developmental changes in perceptions of attractiveness: a role of experience?. Developmental Science.

[CR9] Ewing L, Caulfield F, Read A, Rhodes G (2015). Perceived trustworthiness of faces drives trust behaviour in children. Developmental Science.

[CR10] Ewing, L., Sutherland, C. A. M., & Willis, M. L. (2019). Children show adult-like facial appearance biases when trusting others. *Developmental Psychology*. 10.1037/dev0000747 10.1037/dev0000747 31045400

[CR11] Feldt, L. S., & Kim, S. (2006). Testing the difference between two alpha coefficients with small samples of subjects and raters. *Educational and Psychological Measurement, 66*(4), 589-600. 10.1111/j.1745-3984.2008.00059.x

[CR12] Fisher RA (1915). Frequency distribution of the values of the correlation coefficient in samples of an indefinitely large population. Biometrika..

[CR13] Goldberg LR (1999). A broad-bandwidth, public domain, personality inventory measuring the lower-level facets of several five-factor models. Personality psychology in Europe.

[CR14] Hollis G (2018). Scoring best-worst data in unbalanced many-item designs, with applications to crowdsourcing semantic judgments. Behavior research methods.

[CR15] Jessen S, Grossmann T (2016). Neural and behavioral evidence for infants' sensitivity to the trustworthiness of faces. Journal of Cognitive Neuroscience.

[CR16] Jessen S, Grossmann T (2017). Neural evidence for the subliminal processing of facial trustworthiness in infancy. Neuropsychologia.

[CR17] Kanyongo GY, Brook GP, Kyei-Blankson L, Gocmen G (2007). Reliability and statistical power: How measurement fallibility affects power and required sample sizes for several parametric and nonparametric statistics. Journal of Modern Applied Statistical Methods.

[CR18] Kiritchenko, S., & Mohammad, S. M. (2017). Best-worst scaling more reliable than rating scales: A case study on sentiment intensity annotation. *Proceedings of the Annual Meeting of the Association for Computational Linguistics (ACL), Vancouver, Canada, 2017*.

[CR19] Lipovetsky S, Conklin M (2014). Best-worst scaling in analytical closed-form solution. Journal of Choice Modelling.

[CR20] Louviere, J. J., Flynn, T. N., & Marley, A. A. J. (2015). *Best-worst scaling: Theory, methods and applications*: Cambridge University Press.

[CR21] Ma F, Xu F, Luo X (2016). Children's facial trustworthiness judgments: Agreement and relationship with facial attractiveness. Frontiers in Psychology.

[CR22] McKone, E., Crookes, K., Jeffery, L., & Dilks, D. D. (2012). A critical review of the development of face recognition: Experience is less important than previously believed. *Cognitive neuropsychology, 29*(1-2), 174-212. 10.1080/02643294.2012.660138 10.1080/02643294.2012.660138 22360676

[CR23] Mellor, D., Moore, K. A. (2014). The use of Likert scales with children. *Journal of Pediatric Psychology, 39*(3), 369-379. 10.1093/jpepsy/jst079 10.1093/jpepsy/jst079 24163438

[CR24] Mondloch CJ, Gerada A, Proietti V, Nelson NL (2019). The influence of subtle facial expressions on children's facial first impressions of trustworthiness and dominance is not adult-like. Journal of Experimental Child Psychology.

[CR25] Munafò MR, Nosek BA, Bishop DVM, Button KS, Chambers CD, Percie du Sert N, Ioannidis JPA (2017). A manifesto for reproducible science. Nature Human Behaviour.

[CR26] Olivola CY, Funk F, Todorov A (2014). Social attributions from faces bias human choices. Trends in Cognitive Sciences.

[CR27] Oosterhof NN, Todorov A (2008). The functional basis of face evaluation. Proceedings of the National Academy of Sciences of the United States of America.

[CR28] Open Science Collaboration. (2015). Estimating the reproducibility of psychological science. *Science, 349*(6251). 10.1126/science.aac4716 10.1126/science.aac4716 26315443

[CR29] Paxton JM, Ungar L, Greene JD (2012). Reflection and reasoning in moral judgment. Cognitive Science.

[CR30] R Core Team (2013). R: A language and environment for statistical computing [Computer software manual]. Vienna, Austria. Retrieved from http://www.R-project.org/

[CR31] Sawtooth Software. (2009). Lighthouse Studio. Sequim, WA, United States: Sawtooth Software.

[CR32] Singh D (2004). Mating strategies of young women: Role of physical attractiveness. Journal of sex Research.

[CR33] Todorov A, Olivola CY, Dotsch R, Mende-Siedlecki P (2015). Social attributions from faces: Determinants, consequences, accuracy, and functional significance. Annual Review of Psychology.

[CR34] Trank CQ, Rynes SL, Bretz RD (2002). Attracting applicants in the war for talent: Differences in work preferences among high achievers. Journal of Business and Psychology.

